# Methyl 3-[ferrocen­yl(hydr­oxy)meth­yl]-1-methyl-2′-oxospiro­[pyrrolidine-2,3′-indoline]-3-carboxyl­ate

**DOI:** 10.1107/S1600536809012756

**Published:** 2009-04-18

**Authors:** E. Theboral Sugi Kamala, S. Nirmala, L. Sudha, S. Kathiravan, R. Raghunathan

**Affiliations:** a Department of Physics, Easwari Engineering College, Ramapuram, Chennai 600 089, India; b Department of Physics, SRM University, Ramapuram Campus, Chennai 600 089, India; c Department of Organic Chemistry, University of Madras, Guindy Campus, Chennai 600 025, India

## Abstract

In the title compound, [Fe(C_5_H_5_)(C_20_H_21_N_2_O_4_)], the pyrrolidine ring exhibits an envelope conformation with the spiro-C atom deviating from the plane of the remaining four atoms. The pyrrolidine ring is almost perpendicular to the indolinone ring [dihedral angle = 87.52 (7)°]. The structure is stabilized by an intra­molecular O—H⋯N hydrogen bond and by inter­molecular C—H⋯O and N—H⋯O inter­actions.

## Related literature

For general background to the spiro-indole-pyrrolidine ring system, see: Cordell (1981 ▶). For the biological activity of pyrrolidine-containing compounds and their use in catalysis, see: Witherup *et al.* (1995 ▶); Kravchenko *et al.* (2005 ▶). For the biological activity of oxindole derivatives, see: Glover *et al.* (1998 ▶); Bhattacharya *et al.* (1982 ▶). For puckering and asymmetry parameters, see: Cremer & Pople (1975 ▶); Nardelli (1983 ▶).
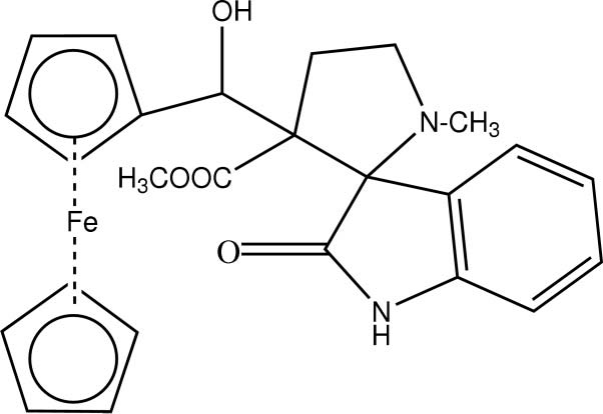


## Experimental

### 

#### Crystal data

[Fe(C_5_H_5_)(C_20_H_21_N_2_O_4_)]
*M* *_r_* = 474.33
Monoclinic, 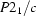

*a* = 9.0120 (2) Å
*b* = 24.0565 (4) Å
*c* = 9.9538 (2) Å
β = 93.2030 (10)°
*V* = 2154.58 (7) Å^3^
*Z* = 4
Mo *K*α radiation
μ = 0.74 mm^−1^
*T* = 293 K
0.25 × 0.25 × 0.20 mm

#### Data collection

Bruker Kappa APEXII diffractometer
Absorption correction: multi-scan (Blessing, 1995 ▶) *T*_min_ = 0.837, *T*_max_ = 0.867
29677 measured reflections
6128 independent reflections
4539 reflections with *I* > 2σ(*I*)
*R*_int_ = 0.036

#### Refinement

*R*[*F* ^2^ > 2σ(*F* ^2^)] = 0.040
*wR*(*F* ^2^) = 0.120
*S* = 1.07
6128 reflections
290 parameters
H-atom parameters constrained
Δρ_max_ = 0.35 e Å^−3^
Δρ_min_ = −0.24 e Å^−3^

### 

Data collection: *APEX2* (Bruker, 2004 ▶); cell refinement: *APEX2* and *SAINT* (Bruker, 2004 ▶); data reduction: *APEX2* and *SAINT*; program(s) used to solve structure: *SIR92* (Altomare *et al.*, 1993 ▶); program(s) used to refine structure: *SHELXL97* (Sheldrick, 2008 ▶); molecular graphics: *ORTEP-3* (Farrugia, 1997 ▶); software used to prepare material for publication: *PLATON* (Spek, 2009 ▶).

## Supplementary Material

Crystal structure: contains datablocks I, global. DOI: 10.1107/S1600536809012756/bt2914sup1.cif

Structure factors: contains datablocks I. DOI: 10.1107/S1600536809012756/bt2914Isup2.hkl

Additional supplementary materials:  crystallographic information; 3D view; checkCIF report

## Figures and Tables

**Table 1:** Hydrogen-bond geometry (Å, °)

*D*—H⋯*A*	*D*—H	H⋯*A*	*D*⋯*A*	*D*—H⋯*A*
O1—H1⋯N1	0.82	2.07	2.781 (2)	145
N2—H2⋯O1^i^	0.86	2.43	3.154 (2)	142
N2—H2⋯O4^i^	0.86	2.52	3.211 (2)	137
C9—H9⋯O4^i^	0.93	2.41	3.185 (2)	141
C2—H2*A*⋯O2	0.97	2.46	2.856 (2)	104
C12—H12⋯O4	0.98	2.41	2.941 (2)	113

Symmetry code: (i) 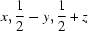.

**Fig. 1.**
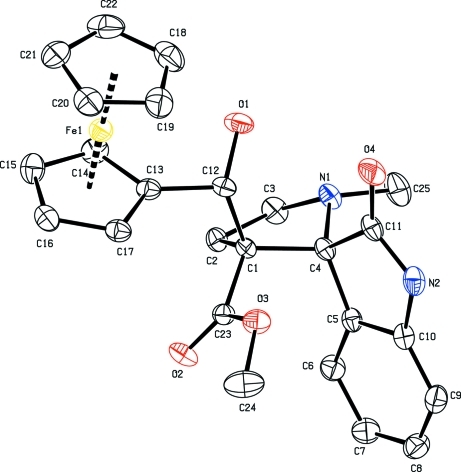
The molecular structure of (I) with 30% probability displacement ellipsoids.

**Fig. 2.**
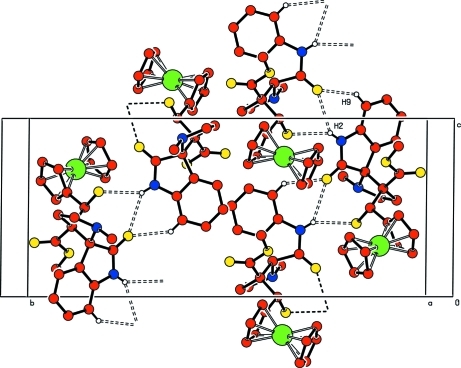
The packing of the molecules viewed down c axis.

## supplementary crystallographic information

### Comment

The spiro-indole-pyrrolidine ring system is a frequently encountered structural motif in many biologically important and pharmacologically relevant alkaloids, such as vincrinstine, vinblastine and spirotypostatins (Cordell, 1981). Pyrrolidine containing compounds are of significant importance because of their biological activities and widespread employment in catalysis (Witherup *et al.*, 1995; Kravchenko *et al.*, 2005). Oxindole derivatives are known to possess a variety of biological activities such as   potent inhibitors of monoamine oxidase (MAO) in human urine and rat tissues (Glover *et al.*, 1998)  and atrial natriuretic peptide-stimulated guanylate cyclase and (iii) a potent antagonist of *in vitro* receptor binding by atrial natriuretic peptide besides possessing a wide range of central nervous system activities (Bhattacharya *et al.*, 1982).

Fig 1 shows the *ORTEP*  plot of compound (I). Bond lengths and angles are comparable with other reported values.

In the molecule the pyrrolidine ring N1/C1/C2/C3/C4 exhibits an *envelope* conformation with envelope on C4 with an assymetry parameter (Nardelli, 1983) ΔC_s_ (C4) = 9.76 (2) and with the puckering parameters (Cremer and Pople, 1975) q2 = 0.4346 (2)Å and φ2 = 150.9 (2)°. The sum of bond angles around N1 [335.72 (5)°] and that around atom N2 [359.68 (2)°] indicate *sp*^3^ and *sp*^2^ hybridizations respectively. The pyrrolidine ring is almost perpendicular to oxyindole ring making a dihedral angle of 87.52 (7)°. The ferrocene ring is perpendicular to both the indole and phenyl rings with dihedral angles of 88.85 (8)° and 88.35 (7)° respectively. In the crystal packing, atoms O1 and O4 are involved in intermolecular and N - H···O interactions and atom O4 also contributes to intermolecular C - H···O interactions.

### Experimental

Experimental procedure A mixture of ferrocenyl Baylis-Hillman adduct, sarcosine and isatin were refluxed in 1,2-dichloethane for 35 h and the solvent was removed under reduced pressure. The crude product was subjected to column chromatography to get the pure product and it was crystallized using slow evaporation technique.

### Refinement

H atoms were placed in idealized positions and allowed to ride on their parent atoms, with C–H = 0.93 or 0.96 Å and U_iso_(H)= 1.2–1.5U_eq_(C).

### Crystal data

**Table:**

[Fe(C_5_H_5_)(C_20_H_21_N_2_O_4_)]	*F*(000) = 992
*M**_r_* = 474.33	*D*_x_ = 1.462 Mg m^−^^3^
Monoclinic, *P*2_1_/*c*	Mo *K*α radiation, λ = 0.71073 Å
Hall symbol: -P 2ybc	Cell parameters from 29677 reflections
*a* = 9.0120 (2) Å	θ = 1.7–29.8°
*b* = 24.0565 (4) Å	µ = 0.74 mm^−^^1^
*c* = 9.9538 (2) Å	*T* = 293 K
β = 93.203 (1)°	Prism, colourless
*V* = 2154.58 (7) Å^3^	0.25 × 0.25 × 0.20 mm
*Z* = 4	

### Data collection

**Table:**

Bruker Kappa APEXII diffractometer	6128 independent reflections
Radiation source: fine-focus sealed tube	4539 reflections with *I* > 2σ(*I*)
graphite	*R*_int_ = 0.036
ω and φ scans	θ_max_ = 29.8°, θ_min_ = 1.7°
Absorption correction: multi-scan (Blessing, 1995)	*h* = −12→12
*T*_min_ = 0.837, *T*_max_ = 0.867	*k* = −32→33
29677 measured reflections	*l* = −13→13

### Refinement

**Table:**

Refinement on *F*^2^	Primary atom site location: structure-invariant direct methods
Least-squares matrix: full	Secondary atom site location: difference Fourier map
*R*[*F*^2^ > 2σ(*F*^2^)] = 0.040	Hydrogen site location: inferred from neighbouring sites
*wR*(*F*^2^) = 0.120	H-atom parameters constrained
*S* = 1.07	*w* = 1/[σ^2^(*F*_o_^2^) + (0.0606*P*)^2^ + 0.4014*P*] where *P* = (*F*_o_^2^ + 2*F*_c_^2^)/3
6128 reflections	(Δ/σ)_max_ = 0.001
290 parameters	Δρ_max_ = 0.35 e Å^−^^3^
0 restraints	Δρ_min_ = −0.24 e Å^−^^3^

### Special details

**Table:**

Geometry. All e.s.d.'s (except the e.s.d. in the dihedral angle between two l.s. planes) are estimated using the full covariance matrix. The cell e.s.d.'s are taken into account individually in the estimation of e.s.d.'s in distances, angles and torsion angles; correlations between e.s.d.'s in cell parameters are only used when they are defined by crystal symmetry. An approximate (isotropic) treatment of cell e.s.d.'s is used for estimating e.s.d.'s involving l.s. planes.
Refinement. Refinement of *F*^2^ against ALL reflections. The weighted *R*-factor *wR* and goodness of fit *S* are based on *F*^2^, conventional *R*-factors *R* are based on *F*, with *F* set to zero for negative *F*^2^. The threshold expression of *F*^2^ > σ(*F*^2^) is used only for calculating *R*-factors(gt) *etc*. and is not relevant to the choice of reflections for refinement. *R*-factors based on *F*^2^ are statistically about twice as large as those based on *F*, and *R*- factors based on ALL data will be even larger.

### Fractional atomic coordinates and isotropic or equivalent isotropic displacement parameters (Å^2^)

**Table:**

	*x*	*y*	*z*	*U*_iso_*/*U*_eq_	
C1	−0.06063 (18)	0.10638 (6)	0.60644 (17)	0.0279 (3)	
C2	0.04940 (19)	0.06715 (7)	0.54079 (18)	0.0335 (4)	
H2A	0.0525	0.0314	0.5858	0.040*	
H2B	0.0201	0.0614	0.4466	0.040*	
C3	0.2003 (2)	0.09546 (8)	0.5554 (2)	0.0414 (4)	
H3A	0.2681	0.0745	0.6152	0.050*	
H3B	0.2433	0.0991	0.4687	0.050*	
C4	0.04647 (19)	0.14248 (6)	0.69968 (18)	0.0298 (3)	
C5	0.08035 (18)	0.11654 (7)	0.83649 (18)	0.0313 (3)	
C6	0.1355 (2)	0.06486 (8)	0.8746 (2)	0.0410 (4)	
H6	0.1624	0.0393	0.8102	0.049*	
C7	0.1499 (2)	0.05180 (9)	1.0103 (2)	0.0482 (5)	
H7	0.1879	0.0173	1.0369	0.058*	
C8	0.1091 (2)	0.08900 (9)	1.1059 (2)	0.0480 (5)	
H8	0.1198	0.0793	1.1963	0.058*	
C9	0.0519 (2)	0.14095 (9)	1.0701 (2)	0.0440 (5)	
H9	0.0224	0.1660	1.1347	0.053*	
C10	0.0408 (2)	0.15379 (7)	0.93552 (19)	0.0351 (4)	
C11	−0.0120 (2)	0.20071 (7)	0.7389 (2)	0.0364 (4)	
C12	−0.14984 (19)	0.14183 (7)	0.49844 (18)	0.0321 (4)	
H12	−0.2047	0.1707	0.5443	0.038*	
C13	−0.25899 (19)	0.10690 (7)	0.41534 (18)	0.0337 (4)	
C14	−0.2432 (2)	0.08509 (9)	0.2844 (2)	0.0428 (4)	
H14	−0.1565	0.0899	0.2303	0.051*	
C15	−0.3741 (2)	0.05584 (9)	0.2448 (2)	0.0475 (5)	
H15	−0.3940	0.0370	0.1584	0.057*	
C16	−0.4723 (2)	0.05901 (8)	0.3502 (2)	0.0429 (4)	
H16	−0.5719	0.0426	0.3500	0.051*	
C17	−0.4023 (2)	0.09065 (8)	0.4551 (2)	0.0372 (4)	
H17	−0.4448	0.0998	0.5408	0.045*	
C18	−0.3799 (3)	0.21945 (10)	0.2619 (3)	0.0662 (7)	
H18	−0.2894	0.2390	0.2941	0.079*	
C19	−0.5077 (3)	0.21230 (9)	0.3337 (3)	0.0574 (6)	
H19	−0.5224	0.2260	0.4247	0.069*	
C20	−0.6115 (3)	0.18195 (9)	0.2516 (3)	0.0518 (5)	
H20	−0.7112	0.1708	0.2756	0.062*	
C21	−0.5473 (3)	0.17064 (10)	0.1296 (2)	0.0577 (6)	
H21	−0.5940	0.1503	0.0532	0.069*	
C22	−0.4038 (3)	0.19409 (12)	0.1361 (3)	0.0670 (7)	
H22	−0.3330	0.1928	0.0650	0.080*	
C23	−0.16990 (19)	0.07551 (7)	0.68956 (17)	0.0323 (4)	
C24	−0.3550 (3)	0.08956 (12)	0.8444 (3)	0.0703 (8)	
H24A	−0.4068	0.1193	0.8860	0.105*	
H24B	−0.3026	0.0678	0.9125	0.105*	
H24C	−0.4251	0.0664	0.7944	0.105*	
C25	0.3000 (2)	0.17773 (10)	0.6751 (3)	0.0585 (6)	
H25A	0.3753	0.1811	0.6111	0.088*	
H25B	0.3378	0.1562	0.7508	0.088*	
H25C	0.2726	0.2141	0.7050	0.088*	
N1	0.17023 (17)	0.15015 (6)	0.61223 (17)	0.0362 (3)	
N2	−0.01332 (18)	0.20279 (6)	0.87400 (17)	0.0410 (4)	
H2	−0.0439	0.2310	0.9177	0.049*	
O1	−0.05131 (15)	0.16789 (6)	0.41099 (14)	0.0456 (3)	
H1	0.0283	0.1742	0.4522	0.068*	
O2	−0.18387 (16)	0.02649 (5)	0.69698 (15)	0.0477 (4)	
O3	−0.25123 (15)	0.11228 (6)	0.75522 (14)	0.0420 (3)	
O4	−0.04452 (17)	0.23784 (5)	0.65992 (16)	0.0496 (4)	
Fe1	−0.42157 (3)	0.136983 (11)	0.28542 (3)	0.03629 (10)	

### Atomic displacement parameters (Å^2^)

**Table:**

	*U*^11^	*U*^22^	*U*^33^	*U*^12^	*U*^13^	*U*^23^
C1	0.0293 (8)	0.0225 (7)	0.0322 (8)	−0.0009 (6)	0.0037 (6)	0.0004 (6)
C2	0.0370 (9)	0.0263 (8)	0.0374 (9)	0.0018 (6)	0.0034 (7)	−0.0049 (7)
C3	0.0367 (10)	0.0372 (10)	0.0512 (11)	0.0043 (7)	0.0111 (8)	−0.0040 (9)
C4	0.0309 (8)	0.0203 (7)	0.0382 (9)	0.0021 (6)	0.0027 (7)	−0.0022 (7)
C5	0.0297 (8)	0.0261 (8)	0.0377 (9)	0.0011 (6)	−0.0016 (7)	−0.0039 (7)
C6	0.0453 (11)	0.0318 (9)	0.0454 (10)	0.0067 (7)	−0.0017 (8)	−0.0005 (8)
C7	0.0533 (12)	0.0405 (11)	0.0499 (12)	0.0062 (9)	−0.0059 (9)	0.0084 (9)
C8	0.0476 (12)	0.0576 (13)	0.0383 (10)	−0.0050 (9)	−0.0032 (9)	0.0040 (10)
C9	0.0426 (11)	0.0494 (11)	0.0400 (10)	−0.0050 (8)	0.0020 (8)	−0.0109 (9)
C10	0.0314 (9)	0.0326 (8)	0.0414 (10)	−0.0014 (7)	0.0007 (7)	−0.0069 (8)
C11	0.0355 (9)	0.0211 (8)	0.0524 (11)	0.0010 (6)	0.0009 (8)	−0.0038 (8)
C12	0.0315 (8)	0.0287 (8)	0.0363 (9)	−0.0004 (6)	0.0037 (7)	0.0074 (7)
C13	0.0307 (9)	0.0346 (9)	0.0356 (9)	0.0019 (7)	0.0017 (7)	0.0065 (7)
C14	0.0374 (10)	0.0497 (11)	0.0421 (10)	0.0043 (8)	0.0082 (8)	−0.0005 (9)
C15	0.0458 (11)	0.0451 (11)	0.0511 (11)	0.0036 (9)	−0.0014 (9)	−0.0106 (10)
C16	0.0389 (10)	0.0351 (9)	0.0541 (12)	−0.0032 (7)	−0.0021 (9)	0.0025 (9)
C17	0.0319 (9)	0.0394 (9)	0.0405 (9)	−0.0002 (7)	0.0044 (7)	0.0065 (8)
C18	0.0576 (15)	0.0477 (13)	0.0903 (19)	−0.0118 (11)	−0.0239 (14)	0.0218 (13)
C19	0.0671 (15)	0.0391 (11)	0.0639 (14)	0.0091 (10)	−0.0138 (12)	0.0007 (11)
C20	0.0417 (11)	0.0452 (11)	0.0672 (14)	0.0054 (9)	−0.0081 (10)	0.0100 (11)
C21	0.0592 (14)	0.0628 (15)	0.0488 (12)	−0.0009 (11)	−0.0174 (11)	0.0125 (11)
C22	0.0619 (15)	0.0744 (17)	0.0647 (16)	0.0001 (13)	0.0020 (12)	0.0339 (14)
C23	0.0319 (9)	0.0329 (9)	0.0319 (8)	−0.0022 (6)	−0.0001 (7)	0.0039 (7)
C24	0.0628 (16)	0.090 (2)	0.0613 (15)	0.0076 (14)	0.0341 (13)	0.0170 (14)
C25	0.0379 (11)	0.0578 (13)	0.0799 (17)	−0.0157 (9)	0.0051 (11)	−0.0090 (12)
N1	0.0309 (7)	0.0300 (7)	0.0483 (9)	−0.0050 (6)	0.0069 (6)	−0.0033 (7)
N2	0.0462 (9)	0.0272 (7)	0.0493 (9)	0.0058 (6)	0.0011 (7)	−0.0134 (7)
O1	0.0404 (8)	0.0487 (8)	0.0477 (8)	−0.0081 (6)	0.0035 (6)	0.0195 (7)
O2	0.0529 (8)	0.0307 (7)	0.0603 (9)	−0.0073 (6)	0.0103 (7)	0.0109 (6)
O3	0.0377 (7)	0.0471 (8)	0.0423 (7)	0.0017 (6)	0.0115 (6)	0.0023 (6)
O4	0.0635 (10)	0.0224 (6)	0.0625 (9)	0.0060 (6)	0.0002 (7)	0.0030 (6)
Fe1	0.03302 (15)	0.03768 (16)	0.03759 (16)	−0.00075 (10)	−0.00312 (10)	0.00460 (11)

### Geometric parameters (Å, °)

**Table:**

C1—C23	1.515 (2)	C15—C16	1.412 (3)
C1—C2	1.541 (2)	C15—Fe1	2.044 (2)
C1—C12	1.560 (2)	C15—H15	0.9800
C1—C4	1.564 (2)	C16—C17	1.412 (3)
C2—C3	1.521 (3)	C16—Fe1	2.0434 (19)
C2—H2A	0.9700	C16—H16	0.9800
C2—H2B	0.9700	C17—Fe1	2.0227 (19)
C3—N1	1.463 (2)	C17—H17	0.9800
C3—H3A	0.9700	C18—C22	1.398 (4)
C3—H3B	0.9700	C18—C19	1.399 (4)
C4—N1	1.464 (2)	C18—Fe1	2.035 (2)
C4—C5	1.513 (3)	C18—H18	0.9800
C4—C11	1.554 (2)	C19—C20	1.411 (3)
C5—C6	1.384 (2)	C19—Fe1	2.039 (2)
C5—C10	1.393 (3)	C19—H19	0.9800
C6—C7	1.385 (3)	C20—C21	1.401 (4)
C6—H6	0.9300	C20—Fe1	2.037 (2)
C7—C8	1.371 (3)	C20—H20	0.9800
C7—H7	0.9300	C21—C22	1.409 (4)
C8—C9	1.390 (3)	C21—Fe1	2.036 (2)
C8—H8	0.9300	C21—H21	0.9800
C9—C10	1.373 (3)	C22—Fe1	2.037 (2)
C9—H9	0.9300	C22—H22	0.9800
C10—N2	1.403 (2)	C23—O2	1.189 (2)
C11—O4	1.215 (2)	C23—O3	1.342 (2)
C11—N2	1.347 (3)	C24—O3	1.433 (3)
C12—O1	1.423 (2)	C24—H24A	0.9600
C12—C13	1.506 (2)	C24—H24B	0.9600
C12—H12	0.9800	C24—H24C	0.9600
C13—C14	1.420 (3)	C25—N1	1.456 (3)
C13—C17	1.426 (3)	C25—H25A	0.9600
C13—Fe1	2.0325 (17)	C25—H25B	0.9600
C14—C15	1.410 (3)	C25—H25C	0.9600
C14—Fe1	2.036 (2)	N2—H2	0.8600
C14—H14	0.9800	O1—H1	0.8200
			
C23—C1—C2	112.62 (14)	C18—C19—H19	126.1
C23—C1—C12	108.57 (13)	C20—C19—H19	126.1
C2—C1—C12	111.21 (14)	Fe1—C19—H19	126.1
C23—C1—C4	110.26 (14)	C21—C20—C19	108.0 (2)
C2—C1—C4	101.62 (13)	C21—C20—Fe1	69.86 (13)
C12—C1—C4	112.49 (13)	C19—C20—Fe1	69.80 (13)
C3—C2—C1	106.16 (13)	C21—C20—H20	126.0
C3—C2—H2A	110.5	C19—C20—H20	126.0
C1—C2—H2A	110.5	Fe1—C20—H20	126.0
C3—C2—H2B	110.5	C20—C21—C22	107.8 (2)
C1—C2—H2B	110.5	C20—C21—Fe1	69.92 (12)
H2A—C2—H2B	108.7	C22—C21—Fe1	69.77 (13)
N1—C3—C2	104.82 (14)	C20—C21—H21	126.1
N1—C3—H3A	110.8	C22—C21—H21	126.1
C2—C3—H3A	110.8	Fe1—C21—H21	126.1
N1—C3—H3B	110.8	C18—C22—C21	108.1 (2)
C2—C3—H3B	110.8	C18—C22—Fe1	69.85 (14)
H3A—C3—H3B	108.9	C21—C22—Fe1	69.76 (13)
N1—C4—C5	117.69 (14)	C18—C22—H22	125.9
N1—C4—C11	108.42 (13)	C21—C22—H22	125.9
C5—C4—C11	101.53 (14)	Fe1—C22—H22	125.9
N1—C4—C1	100.51 (13)	O2—C23—O3	124.13 (17)
C5—C4—C1	113.06 (13)	O2—C23—C1	126.46 (17)
C11—C4—C1	116.27 (14)	O3—C23—C1	109.40 (14)
C6—C5—C10	119.06 (18)	O3—C24—H24A	109.5
C6—C5—C4	131.95 (17)	O3—C24—H24B	109.5
C10—C5—C4	108.95 (15)	H24A—C24—H24B	109.5
C5—C6—C7	118.95 (19)	O3—C24—H24C	109.5
C5—C6—H6	120.5	H24A—C24—H24C	109.5
C7—C6—H6	120.5	H24B—C24—H24C	109.5
C8—C7—C6	120.91 (19)	N1—C25—H25A	109.5
C8—C7—H7	119.5	N1—C25—H25B	109.5
C6—C7—H7	119.5	H25A—C25—H25B	109.5
C7—C8—C9	121.3 (2)	N1—C25—H25C	109.5
C7—C8—H8	119.4	H25A—C25—H25C	109.5
C9—C8—H8	119.4	H25B—C25—H25C	109.5
C10—C9—C8	117.23 (19)	C25—N1—C3	114.47 (16)
C10—C9—H9	121.4	C25—N1—C4	114.98 (16)
C8—C9—H9	121.4	C3—N1—C4	106.27 (13)
C9—C10—C5	122.56 (18)	C11—N2—C10	112.48 (15)
C9—C10—N2	128.26 (18)	C11—N2—H2	123.8
C5—C10—N2	109.15 (17)	C10—N2—H2	123.8
O4—C11—N2	127.13 (17)	C12—O1—H1	109.5
O4—C11—C4	124.98 (18)	C23—O3—C24	116.32 (17)
N2—C11—C4	107.83 (15)	C17—Fe1—C13	41.18 (7)
O1—C12—C13	108.70 (15)	C17—Fe1—C18	128.72 (10)
O1—C12—C1	110.31 (14)	C13—Fe1—C18	106.81 (8)
C13—C12—C1	111.67 (13)	C17—Fe1—C14	68.61 (8)
O1—C12—H12	108.7	C13—Fe1—C14	40.84 (8)
C13—C12—H12	108.7	C18—Fe1—C14	116.51 (10)
C1—C12—H12	108.7	C17—Fe1—C21	150.81 (9)
C14—C13—C17	106.99 (16)	C13—Fe1—C21	167.57 (9)
C14—C13—C12	127.93 (16)	C18—Fe1—C21	67.85 (10)
C17—C13—C12	125.06 (16)	C14—Fe1—C21	130.22 (10)
C14—C13—Fe1	69.71 (11)	C17—Fe1—C22	166.97 (10)
C17—C13—Fe1	69.04 (10)	C13—Fe1—C22	128.40 (9)
C12—C13—Fe1	125.20 (12)	C18—Fe1—C22	40.17 (11)
C15—C14—C13	108.39 (17)	C14—Fe1—C22	108.40 (10)
C15—C14—Fe1	70.06 (12)	C21—Fe1—C22	40.46 (10)
C13—C14—Fe1	69.45 (10)	C17—Fe1—C20	117.57 (9)
C15—C14—H14	125.8	C13—Fe1—C20	149.72 (9)
C13—C14—H14	125.8	C18—Fe1—C20	67.79 (10)
Fe1—C14—H14	125.8	C14—Fe1—C20	168.90 (9)
C14—C15—C16	108.36 (19)	C21—Fe1—C20	40.22 (10)
C14—C15—Fe1	69.48 (12)	C22—Fe1—C20	67.71 (10)
C16—C15—Fe1	69.79 (11)	C17—Fe1—C19	108.01 (9)
C14—C15—H15	125.8	C13—Fe1—C19	115.90 (9)
C16—C15—H15	125.8	C18—Fe1—C19	40.17 (11)
Fe1—C15—H15	125.8	C14—Fe1—C19	148.99 (9)
C15—C16—C17	107.79 (18)	C21—Fe1—C19	67.87 (10)
C15—C16—Fe1	69.80 (12)	C22—Fe1—C19	67.59 (11)
C17—C16—Fe1	68.89 (11)	C20—Fe1—C19	40.51 (9)
C15—C16—H16	126.1	C17—Fe1—C16	40.64 (8)
C17—C16—H16	126.1	C13—Fe1—C16	68.81 (7)
Fe1—C16—H16	126.1	C18—Fe1—C16	167.72 (11)
C16—C17—C13	108.46 (17)	C14—Fe1—C16	68.24 (8)
C16—C17—Fe1	70.47 (11)	C21—Fe1—C16	118.72 (9)
C13—C17—Fe1	69.78 (10)	C22—Fe1—C16	151.33 (11)
C16—C17—H17	125.8	C20—Fe1—C16	109.81 (9)
C13—C17—H17	125.8	C19—Fe1—C16	130.30 (10)
Fe1—C17—H17	125.8	C17—Fe1—C15	68.26 (8)
C22—C18—C19	108.3 (2)	C13—Fe1—C15	68.54 (8)
C22—C18—Fe1	69.98 (15)	C18—Fe1—C15	150.04 (12)
C19—C18—Fe1	70.07 (13)	C14—Fe1—C15	40.45 (8)
C22—C18—H18	125.9	C21—Fe1—C15	110.06 (10)
C19—C18—H18	125.9	C22—Fe1—C15	118.31 (11)
Fe1—C18—H18	125.9	C20—Fe1—C15	131.14 (9)
C18—C19—C20	107.8 (2)	C19—Fe1—C15	169.14 (10)
C18—C19—Fe1	69.77 (14)	C16—Fe1—C15	40.41 (9)
C20—C19—Fe1	69.68 (13)		
			
C23—C1—C2—C3	−138.59 (16)	C12—C13—Fe1—C19	−30.76 (19)
C12—C1—C2—C3	99.27 (17)	C14—C13—Fe1—C16	80.83 (12)
C4—C1—C2—C3	−20.64 (18)	C17—C13—Fe1—C16	−37.55 (11)
C1—C2—C3—N1	−6.0 (2)	C12—C13—Fe1—C16	−156.41 (17)
C23—C1—C4—N1	159.30 (13)	C14—C13—Fe1—C15	37.30 (12)
C2—C1—C4—N1	39.66 (15)	C17—C13—Fe1—C15	−81.07 (12)
C12—C1—C4—N1	−79.34 (15)	C12—C13—Fe1—C15	160.07 (18)
C23—C1—C4—C5	32.95 (18)	C22—C18—Fe1—C17	170.45 (14)
C2—C1—C4—C5	−86.69 (16)	C19—C18—Fe1—C17	−70.40 (17)
C12—C1—C4—C5	154.30 (14)	C22—C18—Fe1—C13	130.39 (15)
C23—C1—C4—C11	−83.95 (18)	C19—C18—Fe1—C13	−110.46 (14)
C2—C1—C4—C11	156.41 (15)	C22—C18—Fe1—C14	87.48 (16)
C12—C1—C4—C11	37.4 (2)	C19—C18—Fe1—C14	−153.37 (13)
N1—C4—C5—C6	−62.1 (3)	C22—C18—Fe1—C21	−37.69 (16)
C11—C4—C5—C6	179.76 (19)	C19—C18—Fe1—C21	81.46 (16)
C1—C4—C5—C6	54.5 (3)	C19—C18—Fe1—C22	119.2 (2)
N1—C4—C5—C10	120.26 (16)	C22—C18—Fe1—C20	−81.28 (16)
C11—C4—C5—C10	2.13 (18)	C19—C18—Fe1—C20	37.87 (15)
C1—C4—C5—C10	−123.17 (15)	C22—C18—Fe1—C19	−119.2 (2)
C10—C5—C6—C7	−0.1 (3)	C22—C18—Fe1—C16	−162.4 (4)
C4—C5—C6—C7	−177.57 (19)	C19—C18—Fe1—C16	−43.2 (5)
C5—C6—C7—C8	0.7 (3)	C22—C18—Fe1—C15	54.3 (2)
C6—C7—C8—C9	0.0 (3)	C19—C18—Fe1—C15	173.49 (16)
C7—C8—C9—C10	−1.2 (3)	C15—C14—Fe1—C17	81.16 (14)
C8—C9—C10—C5	1.8 (3)	C13—C14—Fe1—C17	−38.48 (11)
C8—C9—C10—N2	179.61 (19)	C15—C14—Fe1—C13	119.63 (17)
C6—C5—C10—C9	−1.2 (3)	C15—C14—Fe1—C18	−155.11 (15)
C4—C5—C10—C9	176.81 (17)	C13—C14—Fe1—C18	85.25 (14)
C6—C5—C10—N2	−179.33 (16)	C15—C14—Fe1—C21	−72.58 (17)
C4—C5—C10—N2	−1.3 (2)	C13—C14—Fe1—C21	167.79 (12)
N1—C4—C11—O4	50.5 (2)	C15—C14—Fe1—C22	−112.34 (15)
C5—C4—C11—O4	175.11 (18)	C13—C14—Fe1—C22	128.03 (13)
C1—C4—C11—O4	−61.8 (2)	C15—C14—Fe1—C20	−44.7 (5)
N1—C4—C11—N2	−126.84 (16)	C13—C14—Fe1—C20	−164.3 (4)
C5—C4—C11—N2	−2.24 (18)	C15—C14—Fe1—C19	170.75 (18)
C1—C4—C11—N2	120.90 (17)	C13—C14—Fe1—C19	51.1 (2)
C23—C1—C12—O1	−175.17 (14)	C15—C14—Fe1—C16	37.30 (13)
C2—C1—C12—O1	−50.71 (19)	C13—C14—Fe1—C16	−82.33 (12)
C4—C1—C12—O1	62.52 (18)	C13—C14—Fe1—C15	−119.63 (17)
C23—C1—C12—C13	−54.17 (18)	C20—C21—Fe1—C17	49.7 (3)
C2—C1—C12—C13	70.28 (18)	C22—C21—Fe1—C17	168.43 (19)
C4—C1—C12—C13	−176.49 (14)	C20—C21—Fe1—C13	−148.0 (4)
O1—C12—C13—C14	21.8 (2)	C22—C21—Fe1—C13	−29.3 (5)
C1—C12—C13—C14	−100.2 (2)	C20—C21—Fe1—C18	−81.33 (16)
O1—C12—C13—C17	−156.64 (17)	C22—C21—Fe1—C18	37.43 (18)
C1—C12—C13—C17	81.4 (2)	C20—C21—Fe1—C14	171.99 (13)
O1—C12—C13—Fe1	−68.91 (18)	C22—C21—Fe1—C14	−69.2 (2)
C1—C12—C13—Fe1	169.16 (12)	C20—C21—Fe1—C22	−118.8 (2)
C17—C13—C14—C15	−0.2 (2)	C22—C21—Fe1—C20	118.8 (2)
C12—C13—C14—C15	−178.85 (17)	C20—C21—Fe1—C19	−37.81 (14)
Fe1—C13—C14—C15	−59.44 (14)	C22—C21—Fe1—C19	80.95 (18)
C17—C13—C14—Fe1	59.22 (12)	C20—C21—Fe1—C16	87.17 (15)
C12—C13—C14—Fe1	−119.41 (18)	C22—C21—Fe1—C16	−154.08 (16)
C13—C14—C15—C16	−0.1 (2)	C20—C21—Fe1—C15	130.76 (14)
Fe1—C14—C15—C16	−59.15 (14)	C22—C21—Fe1—C15	−110.48 (17)
C13—C14—C15—Fe1	59.06 (14)	C18—C22—Fe1—C17	−35.1 (5)
C14—C15—C16—C17	0.4 (2)	C21—C22—Fe1—C17	−154.3 (4)
Fe1—C15—C16—C17	−58.59 (13)	C18—C22—Fe1—C13	−68.48 (19)
C14—C15—C16—Fe1	58.96 (15)	C21—C22—Fe1—C13	172.29 (14)
C15—C16—C17—C13	−0.5 (2)	C21—C22—Fe1—C18	−119.2 (2)
Fe1—C16—C17—C13	−59.66 (13)	C18—C22—Fe1—C14	−109.58 (16)
C15—C16—C17—Fe1	59.16 (14)	C21—C22—Fe1—C14	131.19 (16)
C14—C13—C17—C16	0.4 (2)	C18—C22—Fe1—C21	119.2 (2)
C12—C13—C17—C16	179.13 (16)	C18—C22—Fe1—C20	81.51 (17)
Fe1—C13—C17—C16	60.09 (13)	C21—C22—Fe1—C20	−37.72 (15)
C14—C13—C17—Fe1	−59.65 (13)	C18—C22—Fe1—C19	37.54 (16)
C12—C13—C17—Fe1	119.04 (17)	C21—C22—Fe1—C19	−81.69 (17)
C22—C18—C19—C20	0.3 (3)	C18—C22—Fe1—C16	172.29 (18)
Fe1—C18—C19—C20	−59.52 (15)	C21—C22—Fe1—C16	53.1 (3)
C22—C18—C19—Fe1	59.78 (18)	C18—C22—Fe1—C15	−152.56 (15)
C18—C19—C20—C21	−0.1 (3)	C21—C22—Fe1—C15	88.21 (18)
Fe1—C19—C20—C21	−59.65 (16)	C21—C20—Fe1—C17	−155.20 (14)
C18—C19—C20—Fe1	59.57 (16)	C19—C20—Fe1—C17	85.74 (16)
C19—C20—C21—C22	−0.1 (3)	C21—C20—Fe1—C13	166.94 (15)
Fe1—C20—C21—C22	−59.75 (16)	C19—C20—Fe1—C13	47.9 (2)
C19—C20—C21—Fe1	59.61 (15)	C21—C20—Fe1—C18	81.50 (16)
C19—C18—C22—C21	−0.3 (3)	C19—C20—Fe1—C18	−37.55 (16)
Fe1—C18—C22—C21	59.49 (17)	C21—C20—Fe1—C14	−33.5 (5)
C19—C18—C22—Fe1	−59.84 (16)	C19—C20—Fe1—C14	−152.6 (4)
C20—C21—C22—C18	0.3 (3)	C19—C20—Fe1—C21	−119.1 (2)
Fe1—C21—C22—C18	−59.55 (17)	C21—C20—Fe1—C22	37.94 (16)
C20—C21—C22—Fe1	59.85 (16)	C19—C20—Fe1—C22	−81.11 (17)
C2—C1—C23—O2	−6.8 (3)	C21—C20—Fe1—C19	119.1 (2)
C12—C1—C23—O2	116.80 (19)	C21—C20—Fe1—C16	−111.40 (15)
C4—C1—C23—O2	−119.55 (19)	C19—C20—Fe1—C16	129.54 (15)
C2—C1—C23—O3	173.34 (14)	C21—C20—Fe1—C15	−70.86 (18)
C12—C1—C23—O3	−63.05 (17)	C19—C20—Fe1—C15	170.08 (15)
C4—C1—C23—O3	60.60 (17)	C18—C19—Fe1—C17	129.38 (15)
C2—C3—N1—C25	161.08 (18)	C20—C19—Fe1—C17	−111.64 (15)
C2—C3—N1—C4	33.1 (2)	C18—C19—Fe1—C13	85.60 (16)
C5—C4—N1—C25	−50.3 (2)	C20—C19—Fe1—C13	−155.43 (14)
C11—C4—N1—C25	64.0 (2)	C20—C19—Fe1—C18	119.0 (2)
C1—C4—N1—C25	−173.53 (16)	C18—C19—Fe1—C14	51.1 (2)
C5—C4—N1—C3	77.38 (19)	C20—C19—Fe1—C14	170.10 (16)
C11—C4—N1—C3	−168.23 (15)	C18—C19—Fe1—C21	−81.43 (16)
C1—C4—N1—C3	−45.80 (17)	C20—C19—Fe1—C21	37.54 (15)
O4—C11—N2—C10	−175.66 (19)	C18—C19—Fe1—C22	−37.54 (15)
C4—C11—N2—C10	1.6 (2)	C20—C19—Fe1—C22	81.43 (16)
C9—C10—N2—C11	−178.23 (19)	C18—C19—Fe1—C20	−119.0 (2)
C5—C10—N2—C11	−0.2 (2)	C18—C19—Fe1—C16	168.99 (14)
O2—C23—O3—C24	3.4 (3)	C20—C19—Fe1—C16	−72.04 (18)
C1—C23—O3—C24	−176.78 (18)	C18—C19—Fe1—C15	−162.5 (5)
C16—C17—Fe1—C13	−119.26 (16)	C20—C19—Fe1—C15	−43.5 (6)
C16—C17—Fe1—C18	171.42 (14)	C15—C16—Fe1—C17	−119.41 (17)
C13—C17—Fe1—C18	−69.31 (16)	C15—C16—Fe1—C13	−81.38 (13)
C16—C17—Fe1—C14	−81.09 (13)	C17—C16—Fe1—C13	38.03 (11)
C13—C17—Fe1—C14	38.17 (11)	C15—C16—Fe1—C18	−152.6 (4)
C16—C17—Fe1—C21	55.0 (2)	C17—C16—Fe1—C18	−33.2 (5)
C13—C17—Fe1—C21	174.30 (18)	C15—C16—Fe1—C14	−37.34 (12)
C16—C17—Fe1—C22	−160.2 (4)	C17—C16—Fe1—C14	82.07 (12)
C13—C17—Fe1—C22	−41.0 (5)	C15—C16—Fe1—C21	87.70 (15)
C16—C17—Fe1—C20	88.77 (14)	C17—C16—Fe1—C21	−152.89 (13)
C13—C17—Fe1—C20	−151.97 (12)	C15—C16—Fe1—C22	51.4 (2)
C16—C17—Fe1—C19	131.71 (13)	C17—C16—Fe1—C22	170.85 (18)
C13—C17—Fe1—C19	−109.03 (13)	C15—C16—Fe1—C20	130.97 (13)
C13—C17—Fe1—C16	119.26 (16)	C17—C16—Fe1—C20	−109.62 (13)
C16—C17—Fe1—C15	−37.44 (12)	C15—C16—Fe1—C19	172.03 (13)
C13—C17—Fe1—C15	81.82 (12)	C17—C16—Fe1—C19	−68.56 (16)
C14—C13—Fe1—C17	118.38 (16)	C17—C16—Fe1—C15	119.41 (17)
C12—C13—Fe1—C17	−118.9 (2)	C14—C15—Fe1—C17	−82.10 (13)
C14—C13—Fe1—C18	−111.31 (14)	C16—C15—Fe1—C17	37.65 (12)
C17—C13—Fe1—C18	130.31 (14)	C14—C15—Fe1—C13	−37.65 (12)
C12—C13—Fe1—C18	11.45 (19)	C16—C15—Fe1—C13	82.10 (13)
C17—C13—Fe1—C14	−118.38 (16)	C14—C15—Fe1—C18	48.9 (2)
C12—C13—Fe1—C14	122.8 (2)	C16—C15—Fe1—C18	168.69 (17)
C14—C13—Fe1—C21	−48.6 (4)	C16—C15—Fe1—C14	119.75 (18)
C17—C13—Fe1—C21	−167.0 (4)	C14—C15—Fe1—C21	129.14 (14)
C12—C13—Fe1—C21	74.2 (5)	C16—C15—Fe1—C21	−111.11 (14)
C14—C13—Fe1—C22	−72.49 (16)	C14—C15—Fe1—C22	85.47 (15)
C17—C13—Fe1—C22	169.13 (14)	C16—C15—Fe1—C22	−154.78 (13)
C12—C13—Fe1—C22	50.3 (2)	C14—C15—Fe1—C20	169.64 (13)
C14—C13—Fe1—C20	174.09 (16)	C16—C15—Fe1—C20	−70.61 (17)
C17—C13—Fe1—C20	55.7 (2)	C14—C15—Fe1—C19	−153.9 (5)
C12—C13—Fe1—C20	−63.1 (2)	C16—C15—Fe1—C19	−34.2 (5)
C14—C13—Fe1—C19	−153.52 (13)	C14—C15—Fe1—C16	−119.75 (18)
C17—C13—Fe1—C19	88.10 (14)		

### Hydrogen-bond geometry (Å, °)

**Table:**

*D*—H···*A*	*D*—H	H···*A*	*D*···*A*	*D*—H···*A*
O1—H1···N1	0.82	2.07	2.781 (2)	145
N2—H2···O1^i^	0.86	2.43	3.154 (2)	142
N2—H2···O4^ii^	0.86	2.52	3.211 (2)	137
C9—H9···O4^ii^	0.93	2.41	3.185 (2)	141
C2—H2A···O2	0.97	2.46	2.856 (2)	104
C12—H12···O4	0.98	2.41	2.941 (2)	113

Symmetry codes: (i) *x*, −*y*+1/2, *z*+1/2; (ii) .
